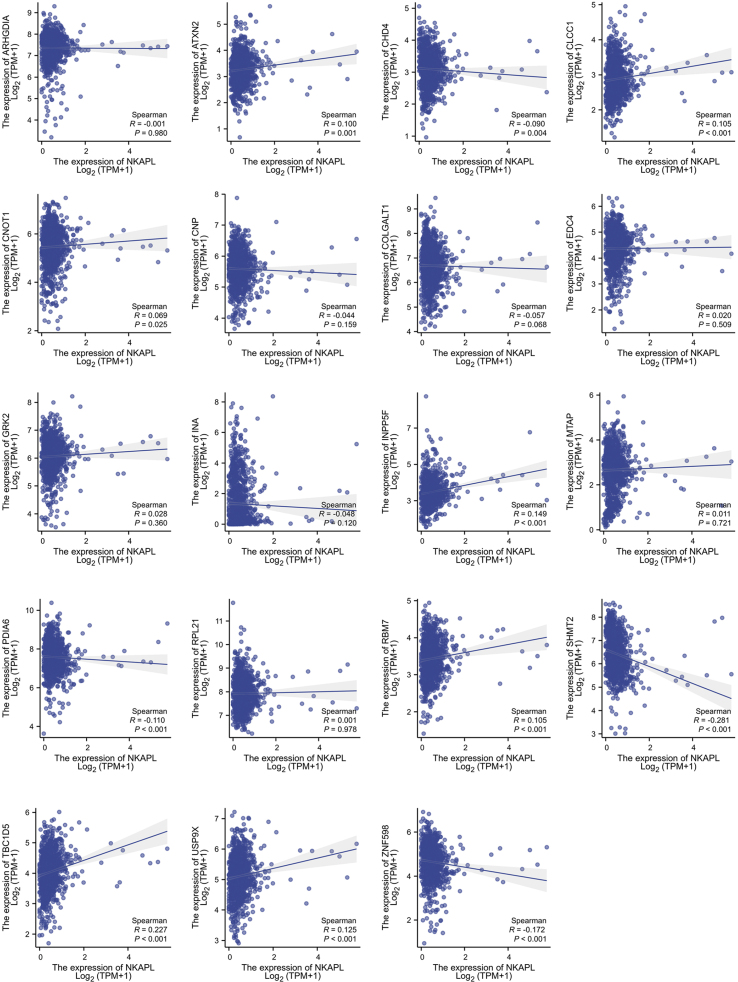# NKAPL suppresses NSCLC progression by enhancing the protein stability of TRIM21 and further inhibiting the NF-κB signaling pathway

**DOI:** 10.1016/j.gendis.2025.101598

**Published:** 2025-03-11

**Authors:** Chunhong Li, Qiang Wang, Fengsheng Dai, Xinni Xiang, Lin Yi, Bianfei Shao, Qian Li, Xi Peng, Renyan Li, Fang Luo, Zhongjun Wu, Tingxiu Xiang

**Affiliations:** a Department of Oncology, The First Affiliated Hospital of Chongqing Medical University, Chongqing 400016, China; b Department of Oncology, Suining Central Hospital, Suining, Sichuan 629000, China; c Chongqing Key Laboratory of Translational Research for Cancer Metastasis and Individualized Treatment, Chongqing University Cancer Hospital, Chongqing University, Chongqing 400030, China; d West China School of Medicine, Sichuan University, Chengdu, Sichuan 610065, China

**Keywords:** Methylation, NF-κB, NKAPL, NSCLC, TRIM21

## Abstract

Non-small cell lung cancer (NSCLC) remains a leading cause of mortality in the clinic. Previous studies have demonstrated that the NF-kappa-B activating protein like (NKAPL) is positively correlated with prognosis in several types of cancers. However, the role of NKAPL in the progression of NSCLC remains unclear. The expression and promoter methylation of NKAPL were examined by real-time PCR, quantitative PCR, and methylation-specific PCR. The functional impacts of NKAPL on NSCLC proliferation were explored by CCK8 assay and colony formation assay. Transwell assay was conducted to investigate the role of NKAPL in NSCLC cell migration and invasion, and the influence on metastasis was verified *in vivo*. Flow cytometry was exploited to analyze the influence on the cell cycle and apoptosis. The regulatory mechanism of NKAPL was investigated by immunoprecipitation-mass spectrometry, western blotting, immunofluorescence, and immunohistochemistry. NKAPL was down-regulated due to promoter methylation, which was associated with poor prognosis in NSCLC patients, while the up-regulation of NKAPL suppressed NSCLC cell proliferation and metastasis both *in vitro* and *in vivo*. Mechanistically, the NF-κB signaling pathway was inhibited because the up-regulation of NKAPL increased the stability and expression of TRIM21. NKAPL suppressed NSCLC cell proliferation and metastasis both *in vitro* and *in vivo* by increasing the stability and expression of TRIM21 and subsequently inhibiting the NF-κB signaling pathway.

## Introduction

According to the global cancer statistics report in 2023, lung cancer has the highest mortality and morbidity.^1^ Non-small cell lung cancer (NSCLC) accounts for more than 80% of all lung cancers, and most NSCLC cases were diagnosed at later stages due to delayed symptom onset and the lack of efficient screening techniques, which results in poor prognosis.^2^, ^3^, ^4^ Therefore, understanding the mechanism of NSCLC progression, invasion, and metastasis, as well as identifying more reliable biomarkers, is critical for NSCLC.

DNA methylation is a prevalent phenomenon in epigenetic regulation and plays a crucial role in early tumorigenesis.^5^ DNA methylation-based liquid biopsy, a noninvasive technique, has significant potential in predicting the progression and outcomes of NSCLC.^6^ In our preliminary studies, multiple methylation markers were identified to be associated with lung cancer prognosis and diagnosis.^7^, ^8^, ^9^ Defining proper combinations of methylation markers provides great chances to improve diagnostic accuracy. According to the importance rank of methylation sites obtained via artificial intelligence machine learning-based analysis of the correlation between lung cancer methylation and clinical characteristics (data source: Genome Atlas Program, TCGA), NF-kappa-B-activating protein-like (NKAPL) has been identified as a potential marker in NSCLC because of its high specificity in distinguishing positive and negative samples.

NKAPL, a protein-encoding gene that is highly conserved in the human genome, exhibits striking homology between mice and humans.^10^ The NKAPL promoter is hypermethylated in liver cancer, which suppresses the expression of NKAPL and indicates a poor prognosis.^11^ Moreover, previous studies have suggested that NKAPL may serve as a prognostic biomarker in triple-negative breast cancer.^12^ In ovarian cancer, NKAPL promoter methylation is implicated in increased platinum resistance.^13^ However, the role of NKAPL in NSCLC progression has not yet been determined.

In this study, the function and clinical value of NKAPL in NSCLC were explored, which indicated that NKAPL was down-regulated in NSCLC because of promoter methylation. Furthermore, ectopic NKAPL inhibited NSCLC cell proliferation, invasion, and migration *in vitro* and *in vivo*. NKAPL also enhanced the stability and expression of tripartite motif-containing 21 (TRIM21), which consequently inhibited the nuclear factor kappa B (NF-κB) signaling pathway. These findings suggest that NKAPL is a potential biomarker and target for NSCLC diagnosis and treatment.

## Materials and methods

### Importance ranking of genes and methylation sites

The importance ranking was processed in the following four steps: i) selecting features, ii) selecting a model, iii) training the model, and iv) ranking importance.

### Feature selection

A dataset obtained from the TCGA database was utilized, and each sample consisted of 485,577 records of methylation sites. To screen informative features, three measurements were applied to the features: i) the *p*-value of a feature between positive and negative populations; ii) the beta-value of a feature between positive and negative populations; and iii) a negative correlation between a feature’s methylation level and the corresponding gene expression level. Only the features that satisfied all three criteria were considered informative.

### Model selection and model training

To find an appropriate classification model for a better understanding of the input features, five widely used machine learning classifiers, namely logistic regression,^14^ support vector machines,^15^ random forest,^16^ the multi-perceptron classifier,^17^ and the Gaussian process classifier^18^ were selected and tested. Models were built and trained by scikit-learn Python.^19^ Each model was trained and validated using 10-fold cross-validation. As a result, the random forest algorithm achieved the highest classification accuracy of 0.9027, hence, it was trained with the full dataset to achieve better performance.

### Importance ranking

Each classifier determines the contributions of each input feature according to a different criterion. For the random forest algorithm, the importance of a feature is measured by the feature’s frequency of being selected as a decision split since the classifier is more likely to select informative features as split nodes. After the importance of each methylation site was obtained, the importance of each gene was measured by summing the importance scores of all the methylation sites on the gene.

### Cell lines and tumor samples

Cell lines (A549, H1299, H358, NCI–H446, NCI–H460, NCI–H2122, NCI-1651, CHAGO-K1, and SW1271) were purchased from the American Type Culture Collection (ATCC; Manassas, VA, USA). The cells were cultured in RPMI 1640 medium (Gibco-BRL, Germany) supplemented with 10% fetal bovine serum (BI, Israel) and 1% penicillin‒streptomycin solution (Gibco-BRL). The cells were incubated in a humid atmosphere with 5% carbon dioxide at 37 °C.

Twenty-six tumor samples and matched adjacent tissue samples from NSCLC patients were collected at Chongqing University Cancer Hospital. Written informed consent was obtained from all patients, and the study was approved by the Ethics Committee of Chongqing University Cancer Hospital (CZLS2022030-A).

### RNA and DNA extraction and PCR analysis

Extraction of RNA and DNA, reverse transcription PCR, and quantitative reverse transcription PCR were conducted according to protocols outlined in a previous study.^20^ The gene expression levels were compared via the 2^−△△CT^ method, and each experiment was conducted in triplicate. β-actin was utilized as an internal control. All primer sequences are displayed in Table 1.

**Table 1:** List of PCR primers used in this study.

Primer	Sequence (5′-3′)	Product size (bp)	Annealing temperature (°C)
NKAPL-F	CGCAGAGCAGATGTTCCTCTTG	200	55/60
NKAPL-R	GTAGCGATATCCACTATACGAG		
NKAPL-m1	ATCGTTTAGCGTTGAGGCGC	102	60
NKAPL-m2	CTCCCCGAAAAACTACGTCG		
NKAPL-u1	TAATTGTTTAGTGTTGAGGTGT	106	60
NKAPL-u2	AACTCCCCAAAAAACTACATCA		
TRIM21-F	AGACACCCAGCAGAGCATACC	114	60
TRIM21-R	CCTGTCACATCTACCTCCCAGTA		
β-actin-F	CTGGAACGGTGAAGGTGACA	139	60
β-actin-R	AGGGACTTCCTGTAACAACGCA		

### Methylation-specific PCR

Genomic DNA samples were extracted from cells and tissues. Methylation-specific PCR was performed according to previous instructions.^8^ The primers utilized in this experiment are listed in Table 1.

### siRNAs, plasmids, and constructed stable cell lines

siRNAs targeting TRIM21 (siTRIM21: 5′-AUUUCCAGGUAUGCUCUGCTT-3′), NKAPL (siNKAPL: 5′-UAUUAUAGGUGCUUCUGGCTT-3′), and negative control siRNAs (siNCs) were synthesized by GenePharma (Shanghai, China). The full-length NKAPL gene was inserted into the pEGFP-C1 plasmid. Transfection was performed via the Lipofectamine 3000 reagent (Carsbad, CA, USA) according to the manufacturer’s protocol. A549 and H460 cells were transfected with the NKAPL plasmid. Stably transfected cells were selected with geneticin (G418).

### Cell counting kit 8 (CCK8)

A 96-well plate was seeded with 1000 cells per well. After 0, 24, 48, 72, and 96 h, the Cell Counting Kit 8 (CCK-8, Beyotime, Jiangsu, China) was used to measure cell viability. Then, the absorbance was measured at 450 nm with a microplate reader (Multislan MK3, Germany). Each experiment was repeated three times.

### Colony formation

Cells were seeded in 6-well plates at 800 cells per well and cultured for 14 days. Surviving colonies (≥50 cells/colony) were counted after fixation and staining with 1% crystal violet. The colony formation rate was calculated using the following formula: colony formation rate = (colony formation numbers/100) × 100%. All the experiments were performed in triplicate.

### Transwell assay

Cells stably expressing NKAPL or the vector were resuspended using a serum-free culture medium. 200 μL μL of serum-free medium with cells (5 × 10^4^ cells for A549 and 6 × 10^4^ cells for H460) was added to the upward side of each chamber. A total of 600 μL medium containing 20% fetal bovine serum was put below each chamber. After incubation (24 h for A549 cells and 48 h for H460 cells), the cells were fixed and stained with 1% crystal violet. Migrated or invasive cells were photographed under a microscope magnification. All assays were performed three times.

### Flow cytometry analysis

Cell cycle arrest and apoptosis were assayed via flow cytometry as described previously.^21^ The data were analyzed with a CELL Quest kit (BD Biosciences). All the experiments were performed in triplicate.

### Hematoxylin-eosin staining

The paraffin sections were processed through routine dewaxing and hydration. Nucleus staining in hematoxylin was performed for 3 min, followed by washing in running tap water for 5 min, submergence in 1% acid ethanol for a few seconds to induce differentiation, and rinsing in running tap water until the cells became blue. The cells were then counterstained in 1% eosin for 10 min. Images were captured under a microscope (Olympus, Japan) after dehydration, clearing, and mounting. All the experiments were performed in triplicate.

### Animal experiments

BALB/c nude mice (4–6 weeks) were purchased from Beijing Vital River Experimental Animal Technology Co., Ltd., Beijing, China. All animal experiments conducted in this study were performed according to protocols approved by the Chongqing Medical University Institutional Animal Care and Use Committee (approval number: IACUC-CQMU-2023-0178).

The impact of NKAPL on the progression of NSCLC was studied by injecting A549 (*n* = 4) and H460 (*n* = 5) cells (5 × 10^6^) cells transfected with the vector or NKAPL plasmid into the left and right flanks of nude mouse subcutaneous xenograft tumors after 4–7 days. A Vernier caliper was used to measure the length and width of the tumors every three days, and the tumor volume was calculated according to the following formula: tumor volume (V) = 0.5 × length × width.^2^ At 18 or 25 days after injection, the mice were euthanized, and the tumors were dissected, weighed, and embedded in paraffin for further examination.

To establish *in vivo* pulmonary metastasis models, BALB/c nude mice (*n* = 3; 4–6 weeks) were injected with A549-Luc (1 × 10^6^) cells transfected with either vector or NKAPL through the caudal vein. Metastatic lesions were monitored weekly via bioluminescence imaging. The mice were anesthetized and injected intraperitoneally with 150 μg of D-luciferin (Yeasen, Shanghai, China) per gram of body weight. After 10 min, the bioluminescence was observed and captured with a PerkinElmer IVIS Lumina system (Caliper Life Sciences, Alameda, California). The fluorescence images were processed with Living Image Software 4.3.1.

### Co-immunoprecipitation and mass spectrometric analysis

The procedures of co-immunoprecipitation can be found in previous instructions.^20^^,^^21^ The antibodies used in this study included anti-GFP (sc-9996; Santa Cruz) and anti-TRIM21 (sc-25351; Santa Cruz) antibodies. Co-immunoprecipitation complexes were detected via SDS‒PAGE and immunoblotting. Co-immunoprecipitation assay was conducted with a GFP antibody. Protein identification of the samples was performed via mass spectrometry (Wayen Biotechnologies, Shanghai, China).

### Immunohistochemistry assay

Immunohistochemistry assays were performed following the protocol outlined in our previous publication.^20^ The sections were incubated with primary antibodies against GFP (sc-9996; Santa Cruz) and Ki67 (sc-23900, 1:200 dilution). The staining results were evaluated by Image-Pro Plus (version 6.0). All the experiments were independently conducted in triplicate.

### Immunofluorescence staining

Immunofluorescence staining was conducted following the methods outlined in our previous study.^20^ Primary antibodies, including anti-GFP (sc-9996; Santa Cruz) and anti-TRIM21 (sc-25351; Santa Cruz), were used for staining. All experiments were independently conducted in triplicate.

### Western blotting

Western blotting was conducted following a previously outlined procedure.^22^ Primary antibodies, including anti-NKAPL (A18845; ABclonal), anti-GFP (sc-9996; Santa Cruz), and anti-β-actin (sc-47778; Santa Cruz) were utilized in this study. An anti–NF–κB pathway antibody sampler kit #9936 (IKKα (3G12), IKKβ (D30C6), anti-phospho-IKKα/β (Ser176/180) (16A6), anti-phospho–NF–κB p65 (Ser536) (93H1), anti-IκBα (L35A5), anti-phospho-IκBα (Ser32) (14D4), anti–NF–κB p65 (D14E12, XP®), and anti-TRIM21 (sc-25351, Santa Cruz) was used.

### Statistical analysis

All the data were analyzed with SPSS 22.0 software (SPSS, Inc., Chicago, IL, USA). Data that followed a normal distribution were analyzed by two-tailed student’s *t*-test. The Mann–Whitney *U* test was employed for data that did not follow a normal distribution. The chi-square test or Fisher’s exact test was used to compare categorical values. *p*-value < 0.05 was recognized as statistically significant.

## Results

### NKAPL was down-regulated in NSCLC and was correlated with clinicopathological parameters

According to the analyses based on artificial intelligence machine learning, NKAPL achieved a high score of 0.9027 in patients with NSCLC (Table S1). First, the expression levels of NKAPL in various cancers were detected, the results of which indicated that NKAPL was down-regulated in various human cancer types (Fig. S1). The expression of NKAPL in both NSCLC cell lines and matched primary NSCLC tissues was evaluated via reverse transcription PCR and quantitative reverse transcription PCR. The results demonstrated that NKAPL expression was down-regulated or silenced in some NSCLC cells (Fig. 1A) and NSCLC tissues from 26 pairs of samples (Fig. 1B). In addition, the expression level of NKAPL also decreased in NSCLC (Fig. 1C, D). The predictive importance of the NKAPL in NSCLC was analyzed via the Kaplan–Meier plotter tool. In patients with NSCLC, longer overall survival was significantly associated with increased NKAPL expression levels (Fig. 1E). The receiver operating characteristic curve for NKAPL levels in the TCGA-NSCLC cohort had an area under the curve of 0.974 (Fig. 1F). Collectively, these data indicated that the down-regulation of NKAPL was crucial in NSCLC progression.

**Figure 1:**
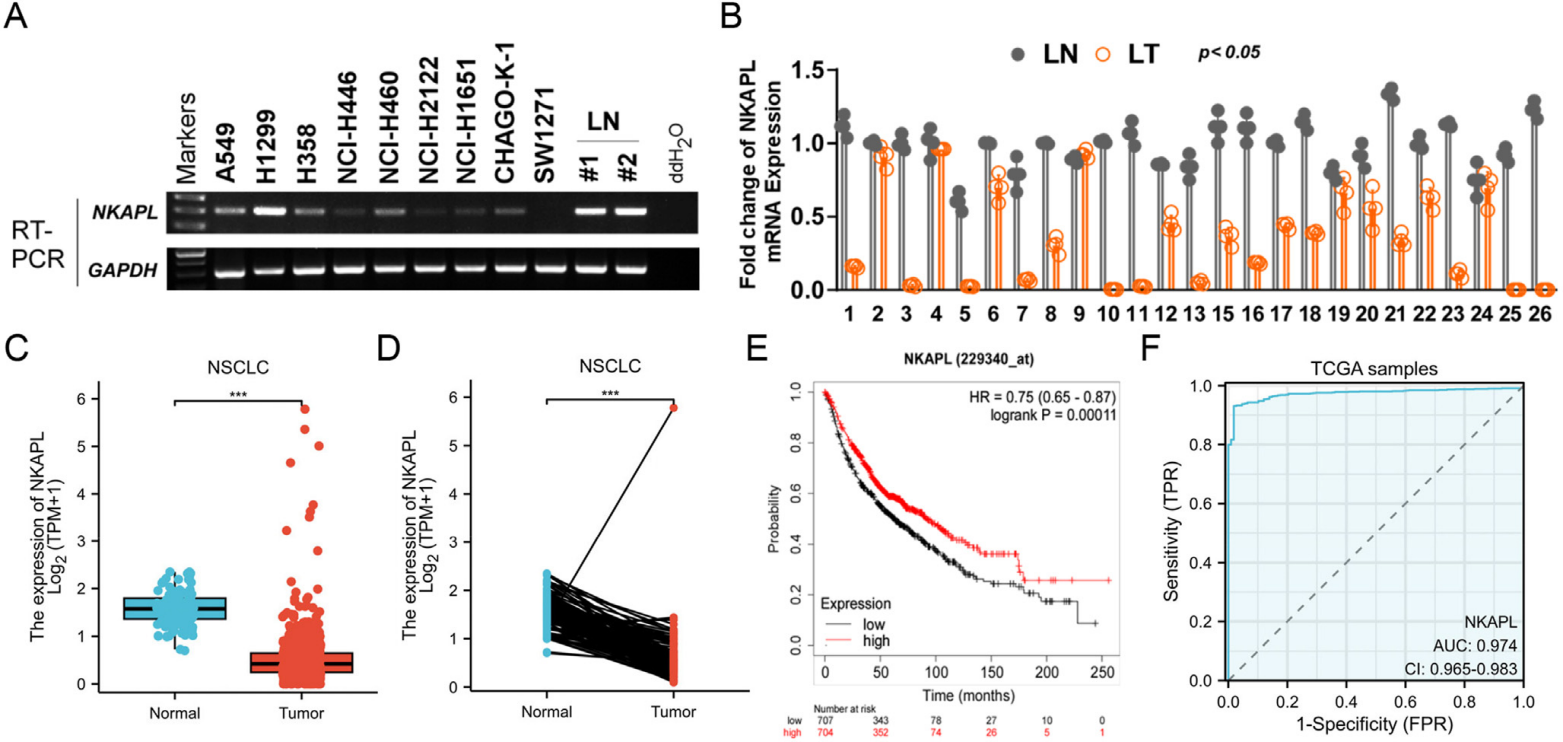
NKAPL is down-regulated in NSCLC tissues and is associated with poor prognosis. **(A)** NKAPL expression levels in human lung cancer cell lines (A549, H1299, H358, NCI–H446, NCI–H460, NCI–H2122, NCI–H1651, CHAGO-K-1, SW1271) and normal lung tissues (#1, #2) were detected via reverse transcription PCR. **(B)** The expression levels of NKAPL in the 26 paired NSCLC tissues were detected via quantitative PCR. LN, normal lung tissues; LT, lung tumor tissues. **(C, D)** The data acquired from the TCGA database presented NKAPL expression in NSCLC. **(E)** Kaplan–Meier analysis of the correlation between NKAPL expression and overall survival in NSCLC patients. **(F)** Receiver operating characteristic curve analyses and area under the curve (AUC) values of NKAPL in NSCLC. ∗*p* < 0.05, ∗∗*p* < 0.01, and ∗∗∗*p* < 0.001.

### The down-regulation of NKAPL in NSCLC was caused by promoter methylation

The regulation of gene expression is significantly impacted by CpG methylation. According to the analysis of the pan-cancer NKAPL methylation status, NKAPL was highly methylated in various tumors, including NSCLC (lung adenocarcinoma and lung squamous cell carcinoma) (Fig. 2A). Hence, a methylation-specific PCR assay was performed to detect the NKAPL promoter methylation status of the 160 NSCLC tissue samples. The results of methylation-specific PCR analysis of primary NSCLC tissues revealed that 99.38 % (159/160) of the NKAPL promoters were hypermethylated (Fig. 2B). In addition, variations were showed in the methylation patterns between the peritumoral lung tissue and the NSCLC tissue samples (Fig. S2), which suggested that promoter methylation of NKAPL was ubiquitous in NSCLC. A549 and H460 cells were then treated with 5-aza-2-deoxycytidine (Aza) for demethylation, with or without the HDAC inhibitor trichostatin A (TSA). The results of quantitative reverse transcription PCR and western blotting assays revealed that the expression of NKAPL was partially restored after treatment (Fig. 2C, D), which supported the idea that the removal of methylation of the promoter region activated NKAPL expression in NSCLC.

**Figure 2:**
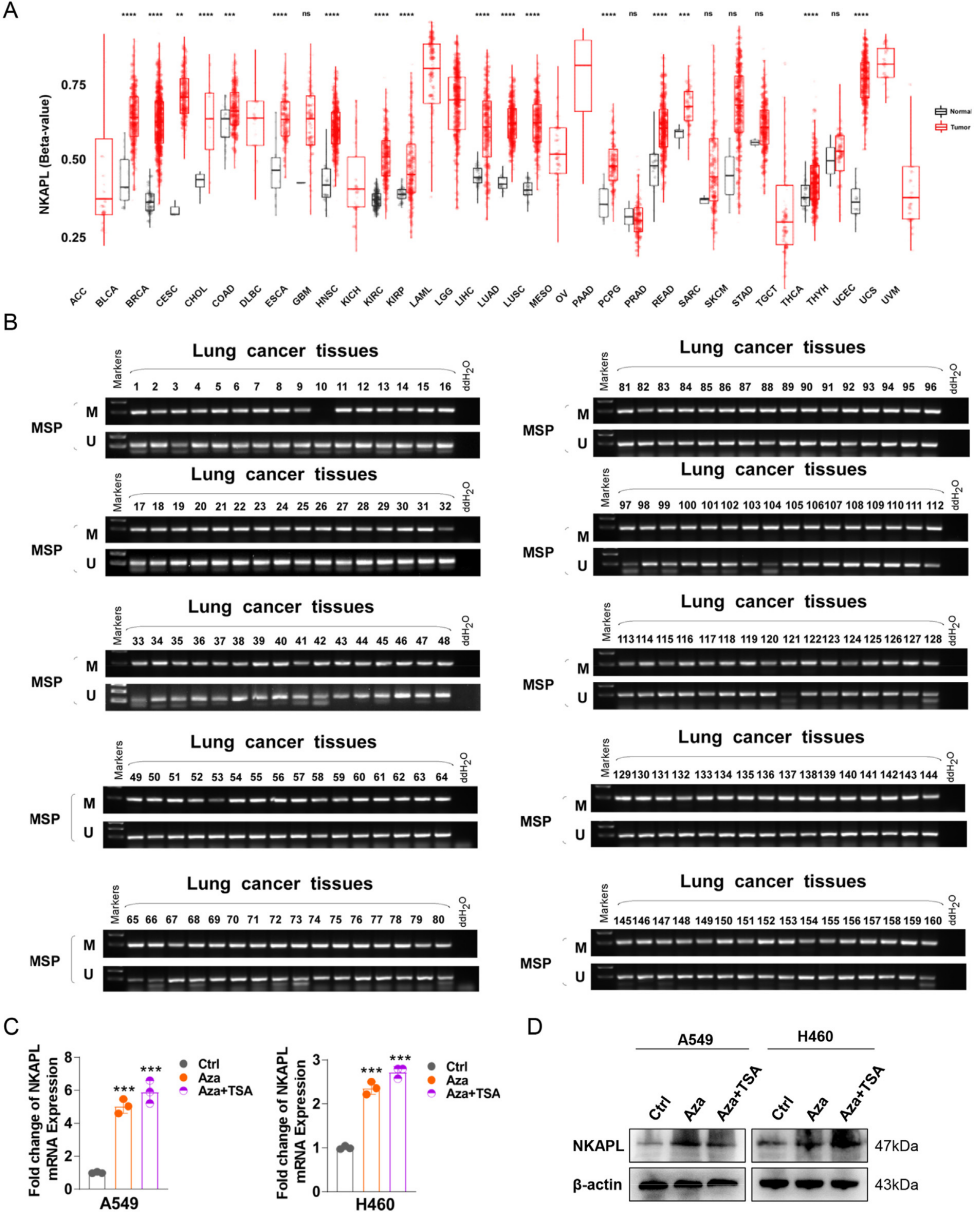
Methylated status of NKAPL in NSCLC tissues. **(A)** DNA methylation levels of NKAPL across cancers. **(B)** Methylation-specific PCR was used to measure NKAPL methylation in primary NSCLC tissues. M, methylated; U, unmethylated. **(C, D)** NKAPL expression in NSCLC cell lines after treatment with 5-aza-2-deoxycytidine (Aza) and trichostatin A (TSA). ∗*p* < 0.05, ∗∗*p* < 0.01, and ∗∗∗*p* < 0.001.

### NKAPL suppressed the proliferation, migration, and invasion of NSCLC cells

To clarify the function of NKAPL in NSCLC, NSCLC cell lines with stable overexpression of NKAPL were constructed (Fig. 3A, B). The corresponding cell proliferation was evaluated by the CCK8 assay and colony formation test, and the results indicated that the overexpression of NKAPL significantly suppressed the proliferation of NSCLC cells (Fig. 3C, D). Furthermore, the influences of NKAPL on the cell cycle and apoptosis were explored via flow cytometry, which revealed that NKAPL induced apoptosis and increased the proportion of cells in the G2/M phase (Fig. 3E, F). The impact of NKAPL on the migration and invasion of NSCLC cells was also investigated via transwell experiments, and the results indicated that the overexpression of NKAPL led to decreased cell migration and invasion, and the differences in migration and invasion capacity between the NKAPL-upregulated group and the control vector group were statistically significant (Fig. 3G, H). In addition, NKAPL expression in H1299 cells was knocked down by siRNA. The results showed that knocking down NKAPL promoted the proliferation, migration, and invasion of H1299 cells (Fig. S3). Taken together, these findings indicated that NKAPL plays a suppressive role in NSCLC.

**Figure 3:**
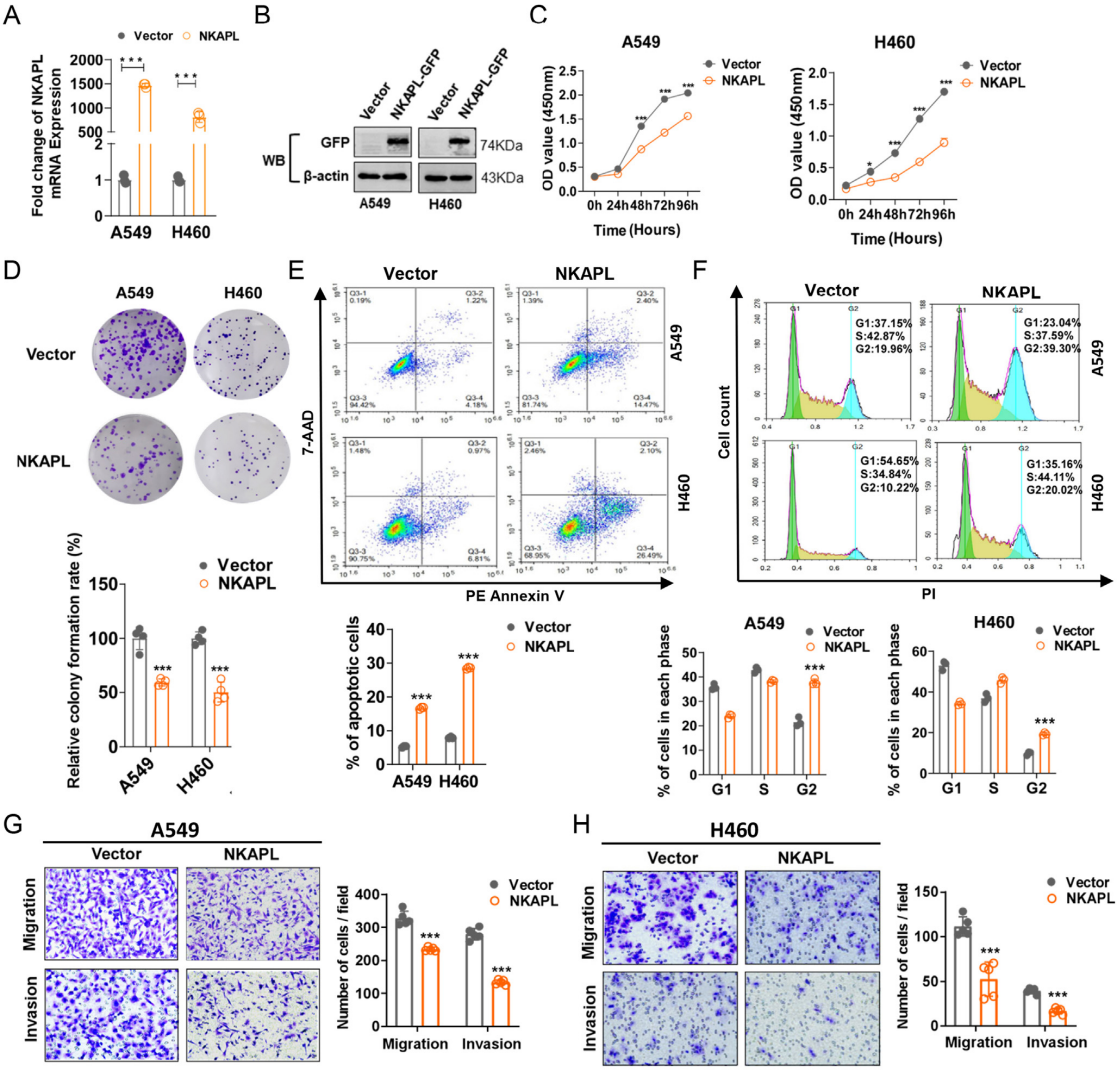
NKAPL suppresses NSCLC cell proliferation, migration, and invasion *in vitro*. **(A, B)** Ectopic NKAPL expression at the transcriptional and posttranscriptional levels was detected by reverse transcription PCR and western blotting. **(C)** The cell proliferation capacity was detected at different time points via CCK8 assays. **(D)** NKAPL overexpression inhibited colony formation. **(E)** The percentage of apoptotic cells was detected by flow cytometry. **(F)** Flow cytometry results of the cell cycle distribution of A549 and H460 cells. **(G, H)** Ectopic NKAPL inhibited the migration and invasion of A549 **(G)** and H460 **(H)** cells, as determined by transwell assays. ∗*p* < 0.05, ∗∗*p* < 0.01, and ∗∗∗*p* < 0.001.

### NKAPL suppressed NSCLC growth and metastasis *in vivo*

We investigated the effects of NKAPL on NSCLC *in vivo* via a xenograft tumor model. The tumor volumes of the xenografts constructed with the NKAPL-overexpressing NSCLC cells were significantly smaller than the volume of those constructed with the control cells (vector) (Fig. 4A, B; Figs. S4A and B), and the NKAPL-overexpressing xenograft tumors weighed less than did those in the vector group (Fig. 4C; Fig. S4C). As expected, immunohistochemistry staining revealed a notable decrease in Ki67 expression in tissues with increased NKAPL expression (Fig. 4D; Fig. S4D).

**Figure 4:**
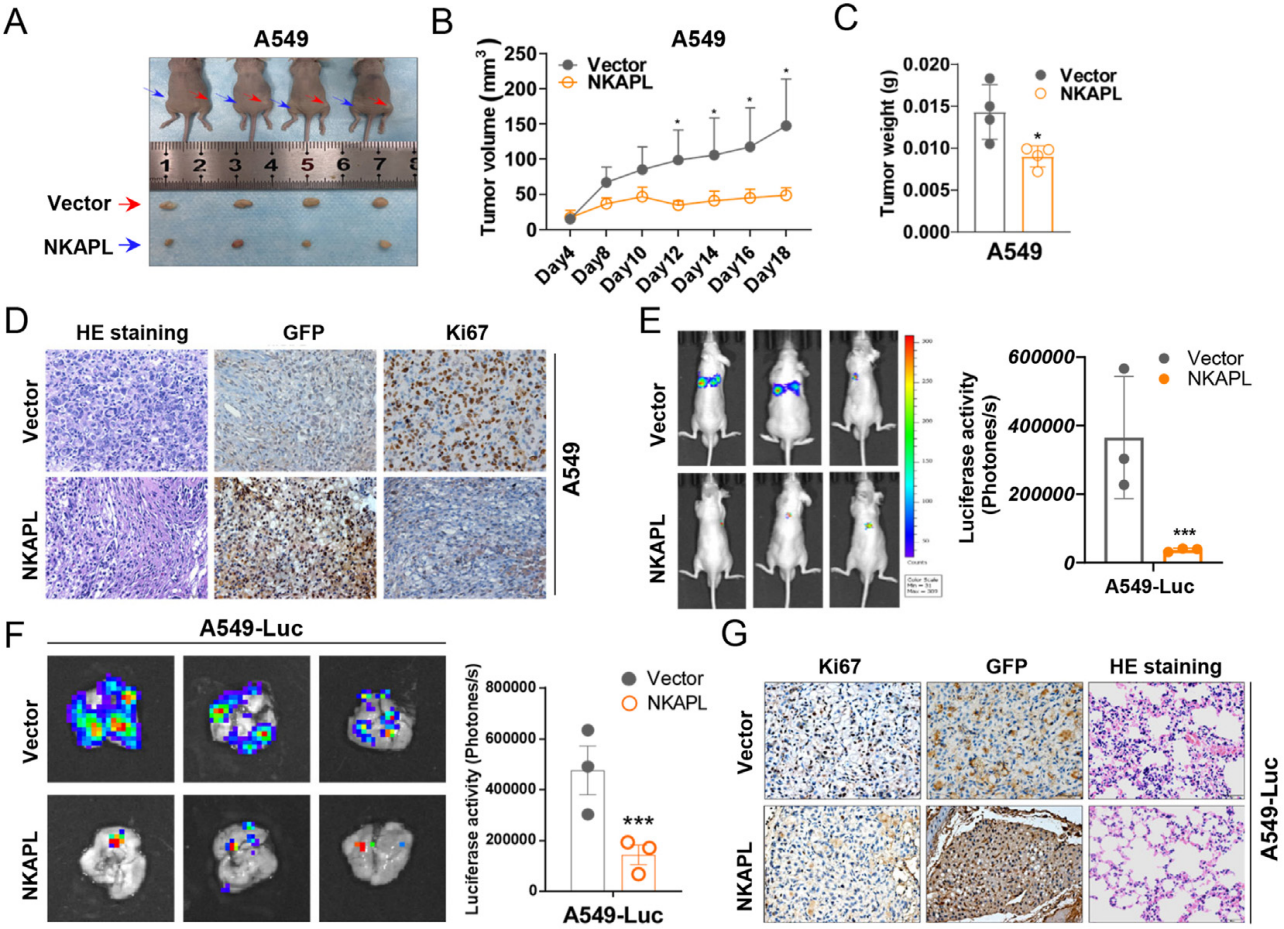
NKAPL suppressed NSCLC growth and metastasis *in vivo*. **(A)** A549 cells stably expressing the vector and NKAPL-GFP were subcutaneously injected into BALB/c nude mice. Images of the subcutaneous tumors of the nude mice were captured. **(B, C)** The volume of the subcutaneous tumors in the nude mice was measured, and the tumors were weighed. **(D)** Hematoxylin-eosin staining and the expression changes of NKAPL and Ki-67 in xenograft tumors were examined by immunohistochemistry staining. **(E, F)** Representative bioluminescence images of the metastatic mouse models at the indicated times imaged by an *in vivo* imaging system. **(G)** Hematoxylin-eosin-stained sections showed pulmonary metastatic foci indicating A549-Luc cells in lung tissue sections. The data were presented as mean ± standard deviation (*n* = 3). ∗*p* < 0.05, ∗∗*p* < 0.01, and ∗∗∗*p* < 0.001.

**Figure 5:**
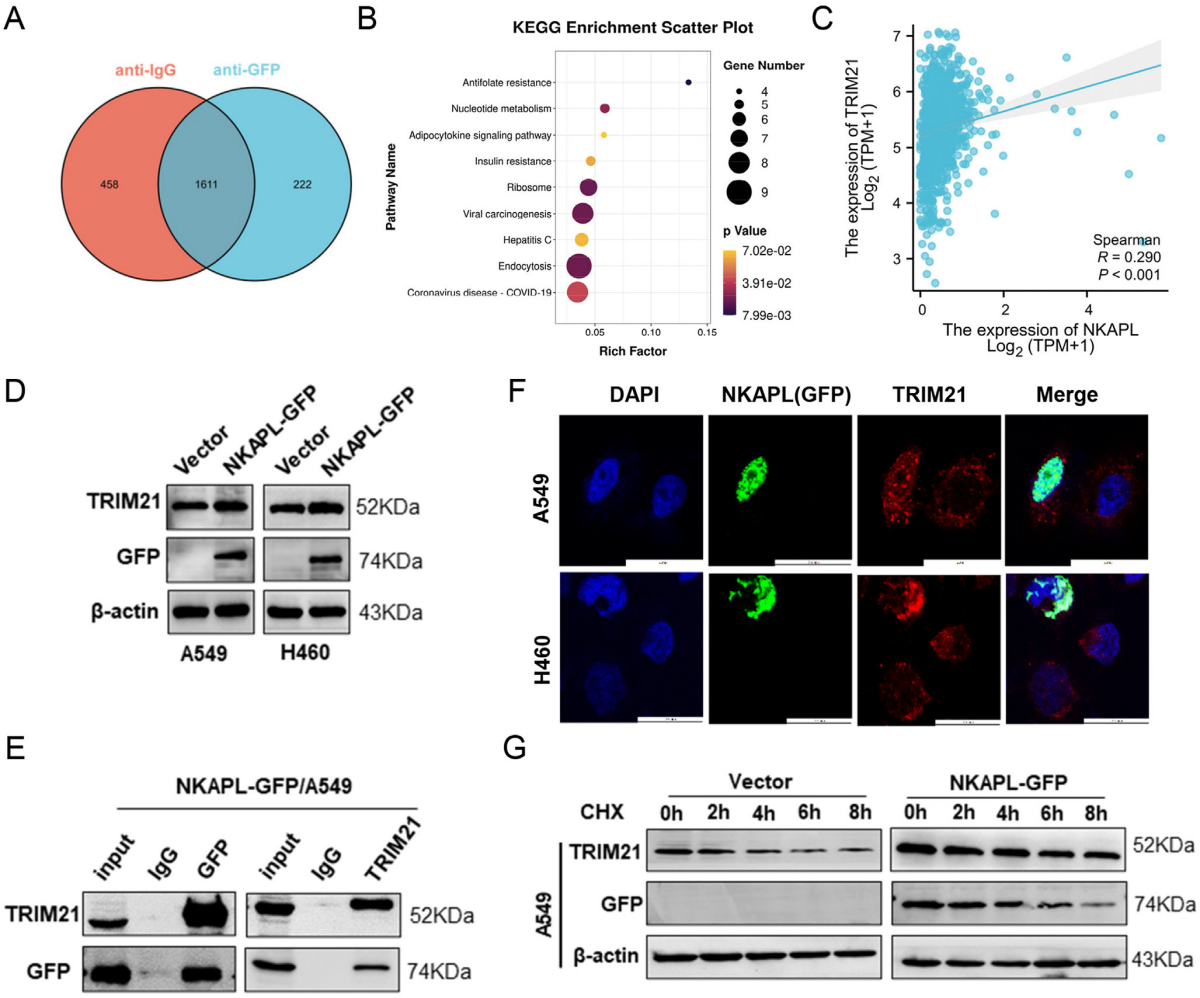
NKAPL interacts with the TRIM21 protein and increases its stability. **(A)** The Venn diagram illustrating the number of both specific and nonspecific NKAPL interactors. **(B)** KEGG pathway analysis of NKAPL-interacting proteins. **(C)** Correlation analysis of NKAPL and TRIM21 mRNA levels in NSCLC tissues. **(D)** The levels of TRIM21 were determined by western blotting analysis when NKAPL was overexpressed. **(E)** Co-immunoprecipitation-western blot analysis of A549 cells. The lysates were incubated with anti-GFP antibodies, and the fractionated immunoprecipitates were examined via western blotting with antibodies against TRIM21 and GFP. **(F)** A549 and H460 cells were transfected with a GFP-tagged NKAPL overexpression plasmid for 48 h. Immunofluorescence staining was performed using anti-TRIM21 antibodies, and the cell nuclei were stained with DAPI (blue). The subcellular localization of GFP (green) and TRIM21 (red) was observed via laser scanning confocal microscopy. **(G)** A549 cells were transfected with NKAPL-GFP for 48 h and then treated with the protein synthesis inhibitor cycloheximide (CHX; 100 μg/mL) for 0, 2, 4, 6, or 8 h. The expression levels of NKAPL and TRIM21 were measured via western blotting.

To study the impact of NKAPL on tumor metastasis *in vivo*, NKAPL-overexpressing A549-Luc cells were intravenously injected through the caudal vein to establish a metastatic mouse model. As shown in the *in vivo* fluorescence imaging results (Fig. 4E, F), the overexpression of NKAPL significantly reduced the fluorescence intensity of metastatic cells in the lung, which was per the results of tumor hematoxylin-eosin staining (Fig. 5G). Therefore, NKAPL expression was negatively correlated with the metastatic capacity of NSCLC *in vivo*.

### NKAPL could interact with the TRIM21 protein and improve its protein stability

To determine the mechanism(s) underlying the suppressive effects of NKAPL on NSCLC progression, co-immunoprecipitation and mass spectrometry were performed in A549 cells. A total of 222 proteins were identified as NKAPL-binding proteins (Fig. 5A and Table S2). KEGG pathway enrichment analysis suggested that the coding genes that corresponded to the identified proteins were enriched mainly in the endocytosis, viral carcinogenesis, and ribosome pathways in cancer, which are intimately associated with cancer progression (Fig. 5B). To further identify the downstream target of NKAPL, bioinformatics analysis was performed to investigate the relationships between NKAPL expression and the top 20 proteins interacting with NKAPL, and the results demonstrated that TRIM21 had the highest correlation with NKAPL expression in NSCLC (Fig. 5C; Fig. S5). Western blotting and immunofluorescence analyses revealed that NKAPL overexpression up-regulated TRIM21 protein expression in A549 and H460 cells (Fig. 5D‒F). In addition, co-immunoprecipitation experiments confirmed the interaction between NKAPL and TRIM21 proteins (Fig. 5E). Furthermore, NKAPL overexpression prolonged the half-life of TRIM21 protein (Fig. 5G). These findings indicated that NKAPL could enhance TRIM21 expression by increasing protein stability.

### NKAPL inhibited the NF-κB signaling pathway through TRIM21

**Figure 6:**
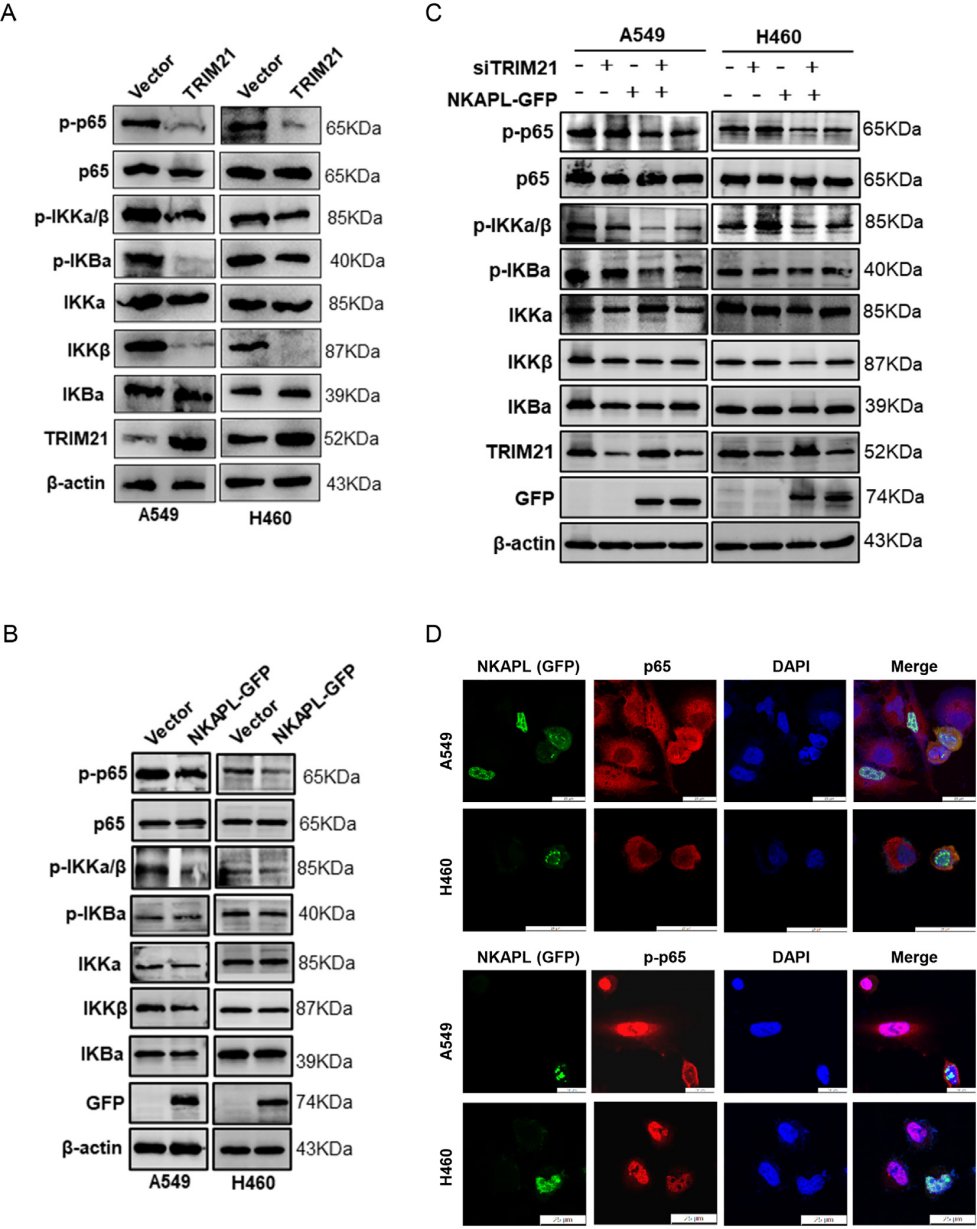
NKAPL inhibits the NF-κB signaling pathway through TRIM21. **(A**–**C)** Western blotting analysis of the expression of phosphorylated p65, p65, phosphorylated IKKα/β, phosphorylated IKBα, IKKα, IKKβ, and IKBα in (A) TRIM21 overexpression, (B) NKAPL-GFP overexpression, and (C) NKAPL-GFP-overexpression plus TRIM21 knockdown. **(D)** Immunofluorescence staining was performed with anti-p65 and anti-phosphorylated p65 antibodies, and the cell nuclei were stained with DAPI (blue). Subcellular localizations of GFP (green), p65 (red), and phosphorylated p65 (red) were observed via laser scanning confocal microscopy.

Some studies focusing on non-threatening illnesses have indicated that TRIM21 stimulates p65, leading to the activation of the NF-κB signaling pathway and the onset of an inflammatory reaction.^23^, ^24^, ^25^ However, whether TRIM21 can regulate the NF-κB signaling pathway in NSCLC has not been reported. Therefore, we first investigated the effect of TRIM21 on the NF-κB signaling pathway in NSCLC cells. The western blotting results indicated that TRIM21 overexpression led to a decrease in p-p65 expression, along with decreased expression levels of IKKβ and p-IKKα/β (Fig. 6A). Specifically, NKAPL overexpression also led to a decrease in p-p65 expression, along with a reduction in the levels of IKKβ and p-IKKα/β (Fig. 6B). Moreover, the protein levels of p-p65 and p-IKKα/β were rescued by knocking down TRIM21 in stable NKAPL-expressing A549 and H460 cells (Fig. 6C). The phosphorylation of p65 and its nuclear translocation are essential for NF-κB activation.^26^ Therefore, the effects of NKAPL on p65 and p-p65 expression and localization were examined. The results revealed that the overexpression of NKAPL could decrease p-p65 and nuclear entry, but p65 was not affected by these regulatory effects (Fig. 6D). Taken together, these results verified that NKAPL inhibits the NF-κB signaling pathway by increasing TRIM21 protein expression.

### TRIM21 knockdown mitigated the effects of NKAPL on NSCLC cell phenotypes

**Figure 7:**
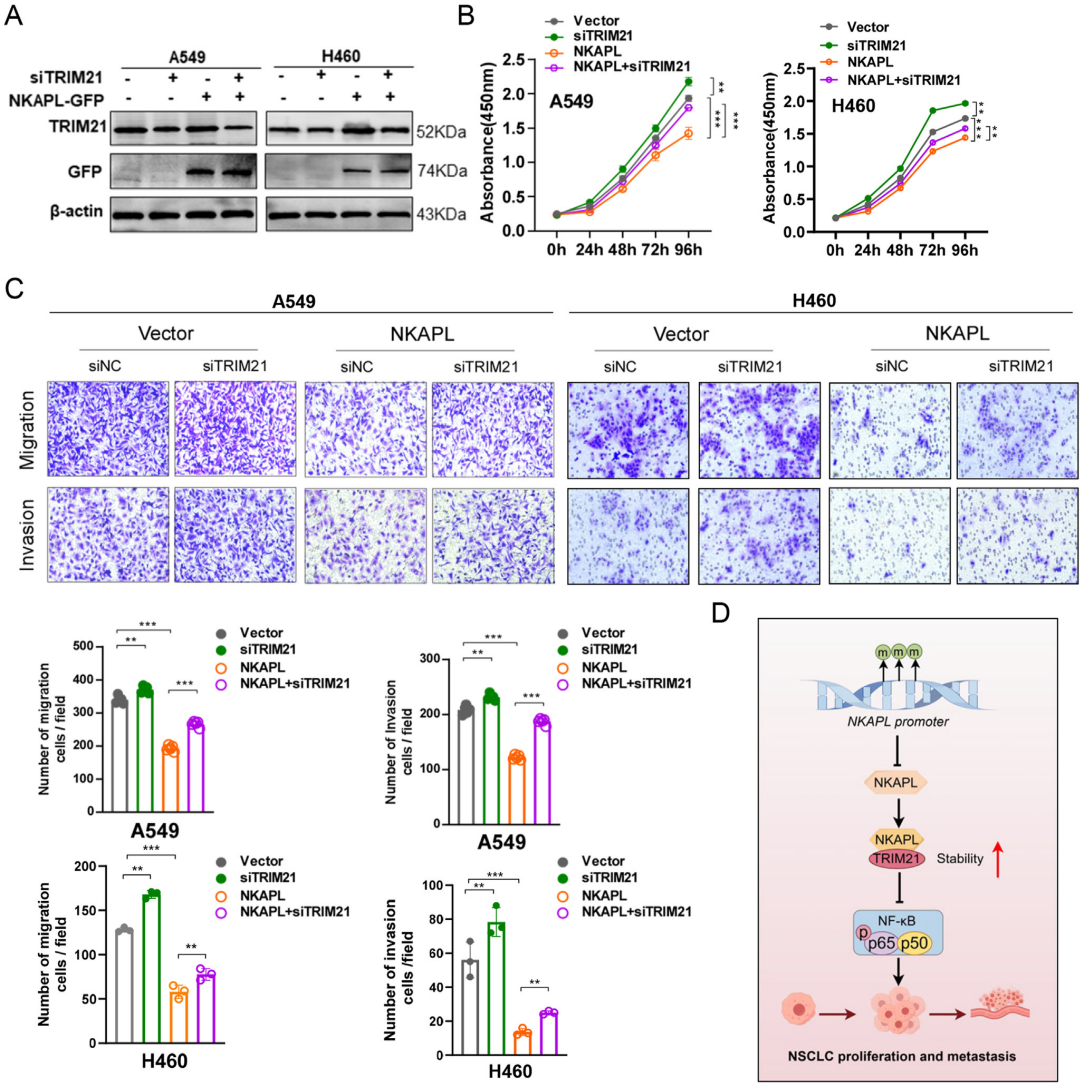
TRIM21 knockdown mitigates the effects of NKAPL on NSCLC cell phenotypes. **(A)** A549 and H460 cells were transfected with si-TRIM21 or co-transfected with si-TRIM21 and NKAPL-GFP overexpression plasmids. The expression level of TRIM21 was verified by western blotting. **(B)** Cell proliferation was determined by a CCK8 assay. **(C)** Changes in cell migration and invasion capacity were evaluated via transwell assays. **(D)** Schematic illustration of the regulation of NSCLC malignant progression by NKAPL. The data were presented as mean ± standard deviation (*n* = 3). ∗∗*p* < 0.01 and ∗∗∗*p* < 0.001.

To determine whether NKAPL reduced NSCLC cell malignancy by up-regulating TRIM21, rescue experiments were conducted. A549 and H460 cells were co-transfected with the NKAPL overexpression plasmid and siRNAs targeting TRIM21 (Fig. 7A). CCK8 assays revealed that TRIM21 down-regulation reversed the inhibitory effect of NKAPL on the proliferation of NSCLC cells (Fig. 7B). Transwell assays revealed that TRIM21 down-regulation reversed the suppressive effects of NKAPL on the invasion and migration of NSCLC cells (Fig. 7C). These results demonstrated that TRIM21 was the key molecule involved in the process by which NKAPL inhibited NF-κB signaling in NSCLC.

## Discussion

Investigating the potential mechanisms underlying NSCLC growth and identifying new diagnostic and therapeutic targets have become a current research focus.^27^ DNA methylation is a common early event in carcinogenesis and has significant advantages as a diagnostic and screening biomarker for tumors.^28^, ^29^, ^30^, ^31^ The deep mining of methylation profiles was performed to identify the optimal methylation gene combinations for enhancing diagnostic accuracy.

In NSCLC, we found that NKAPL achieved a high importance score, which was calculated by summing the importance scores of all the methylation sites on the gene. We also revealed that NKAPL was down-regulated in NSCLC due to promoter methylation. As a protein-coding gene, NKAPL is highly polymorphic and located on chromosome 6p22.1.^32^^,^^33^ It plays a pivotal role in the migration of embryonic cortical neurons, affecting cognitive function during early brain development in early-onset schizophrenia patients. Large-scale data analysis has revealed that the NKAPL gene is a susceptibility locus for rheumatoid arthritis.^10^^,^^33^^,^^34^ Notably, NKAPL methylation and expression levels serve as crucial markers for the diagnosis and prognosis prediction of triple-negative breast cancer patients.^12^ Furthermore, abnormal methylation of NKAPL has been shown to be associated with increased acquired platinum resistance in high-grade serous ovarian cancer.^13^ Conversely, in liver cancer, promoter hypermethylation-induced low expression of NKAPL results in a poor prognosis.^11^ Nevertheless, no study focused on the functions of NKAPL in NSCLC has been reported yet.

Subsequent *in vitro* experiments confirmed that increased NKAPL expression in NSCLC cell lines resulted in the inhibition of cell proliferation, decreased migration and invasion, cell cycle arrest at the G2/M phase, and increased programmed cell death. The *in vivo* experiments indicated that the overexpression of NKAPL substantially inhibited tumor growth and metastasis in the metastatic mouse models. Collectively, the data presented above illustrate the role of NKAPL as a newly verified tumor suppressor in NSCLC.

To further investigate the mechanism of NKAPL in NSCLC, immunoprecipitation-mass spectrometry analysis was conducted. Among the 222 identified interacting proteins, a pivotal downstream target gene, TRIM21, was highlighted based on the high importance score calculated based on a correlation analysis as well as an extensive review of the literature. Moreover, co-immunoprecipitation analyses revealed a significant association between TRIM21 and NKAPL. The western blotting and immunofluorescence results indicated that the overexpression of NKAPL substantially elevated the protein level of TRIM21. Additionally, NKAPL plays a pivotal role in enhancing the stability of TRIM21. Previous studies have shown that TRIM21 enhances IKKβ ubiquitination, leading to the suppression of the NF-κB signaling pathway.^35^ Interestingly, some studies focusing on non-threatening illnesses have indicated that TRIM21 stimulates p65, leading to the activation of the NF-κB signaling pathway and the onset of an inflammatory reaction.^23^, ^24^, ^25^ In this study, when TRIM21 was knocked down, p65 phosphorylation was partially restored by NKAPL overexpression, which highlighted the ability of NKAPL to suppress the NF-κB signaling pathway via TRIM21. NF-κB serves as a crucial transcription factor for governing inflammatory signaling cascades, and it is intimately intertwined with the tumor-associated inflammatory milieu.^36^, ^37^, ^38^ The NF-κB signaling pathway is essential for the development of cancer, tumor cell proliferation and invasion, and the formation of new blood vessels.^39^ These findings align with numerous well-documented studies that link the NF-κB pathway to tumor development.^37^^,^^39^

TRIM21 is a multifaceted protein.^40^^,^^41^ Recent evidence has implicated TRIM21 in various activities in cancers.^42^ Notably, TRIM21 plays a tumor-suppressive role in specific cancer types, including but not limited to breast, renal, and colorectal cancer.^43^, ^44^, ^45^, ^46^, ^47^ Paradoxically, it has also been reported to promote tumorigenesis in other cancer types, such as brain and liver cancer.^48^^,^^49^ However, the precise role and intricate mechanisms of TRIM21 in cancers remain unclear. Here, we found that TRIM21 could inhibit the proliferation, migration, and invasion of NSCLC cells. Furthermore, the knockdown of TRIM21 reversed the inhibitory effect of NKAPL on NSCLC cells.

In the future, we will continue to focus on the regulatory mechanism of TRIM21 in NKAPL and develop NKAPL methylation inhibitors and NKAPL-targeted delivery systems for combination therapy for NSCLC.

## Conclusions

In NSCLC, the NKAPL level is reduced due to promoter methylation, and it suppresses NSCLC proliferation and metastasis both *in vitro* and *in vivo*. NKAPL enhances the stability and expression of TRIM21, which consequently inhibits the NF-κB signaling pathway (Fig. 7D). Furthermore, low expression of NKAPL can be recognized as an important marker for poor prognosis in NSCLC patients. These findings provide new insights into the progression of NSCLC and indicate the potential of NKAPL as a biomarker and therapeutic target for NSCLC.

## CRediT authorship contribution statement

**Chunhong Li:** Writing – original draft, Visualization, Validation, Software, Methodology, Investigation, Data curation. **Qiang Wang:** Writing – original draft, Software, Resources, Methodology, Investigation, Data curation. **Fengsheng Dai:** Writing – original draft, Validation, Software, Resources, Formal analysis. **Xinni Xiang:** Resources. **Lin Yi:** Project administration, Methodology, Investigation. **Bianfei Shao:** Validation, Supervision, Software, Data curation. **Qian Li:** Validation, Supervision, Software, Methodology. **Xi Peng:** Software, Resources, Methodology. **Renyan Li:** Validation, Software, Formal analysis, Data curation. **Fang Luo:** Writing – review & editing, Visualization, Validation, Supervision, Project administration, Formal analysis, Data curation. **Zhongjun Wu:** Writing – review & editing, Visualization, Validation, Supervision, Project administration, Funding acquisition, Conceptualization. **Tingxiu Xiang:** Writing – review & editing, Supervision, Project administration, Funding acquisition, Formal analysis, Data curation, Conceptualization.

## Ethics declaration

Pathologists performed histological diagnoses of the clinical samples, and patients provided informed consent for the collection of tissue samples. This study was approved by the Ethics Committee of Chongqing University Cancer Hospital (approval notices: CZLS2022030-A). The Laboratory Animal Center of Chongqing Medical University conducted all the experiments involving animals (IACUC-CQMU-2023-0178).

## Data availability

Some of the data that support the findings of this study are not openly available for reasons of sensitivity and are available from the corresponding author upon reasonable request.

## Funding

This study was supported by the 10.13039/501100001809National Natural Science Foundation of China (No. 82172619), the 10.13039/501100005230Natural Science Foundation of Chongqing, China (No. CSTC2021jscx-gksb-N0023, CSTB2022NSCQ-M SX0063, CSTB2023NSCQ-MSX0386), the Medical and Industrial Integration Project (No. 2022CDJYGRH-002), and Sichuan Science and Technology Program, China (No. 2025ZNSFSC1926).

## Conflict of interests

The authors declared no potential conflict of interests.

## Figures and Tables

**figs1:**
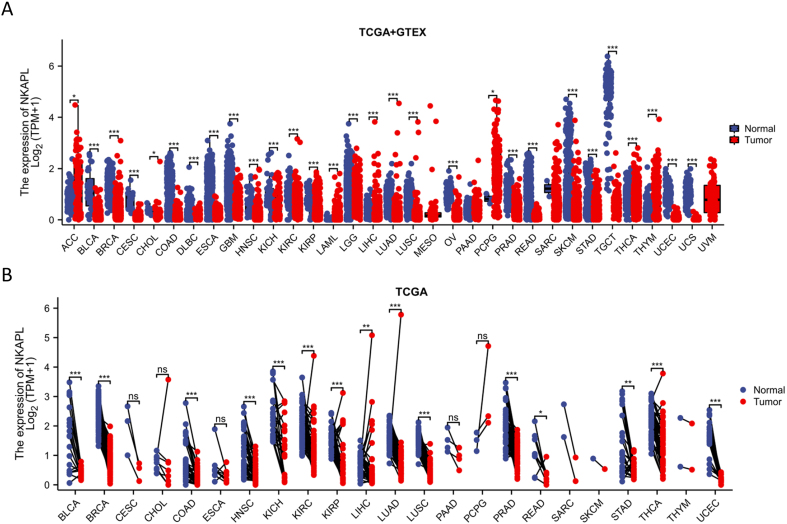


**figs2:**
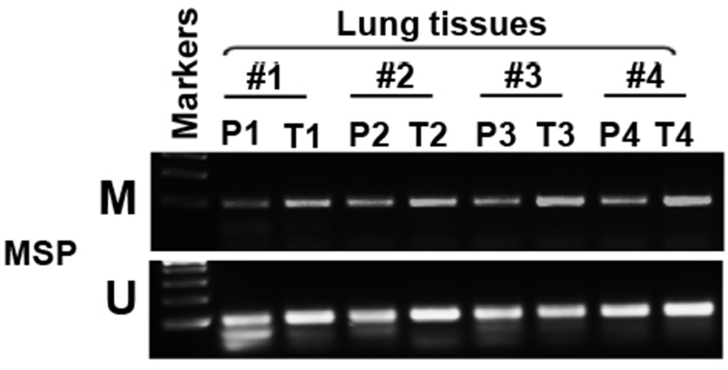


**figs3:**
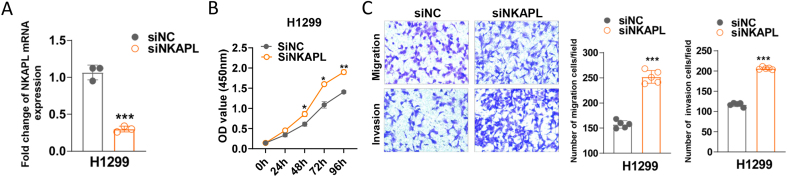


**figs4:**
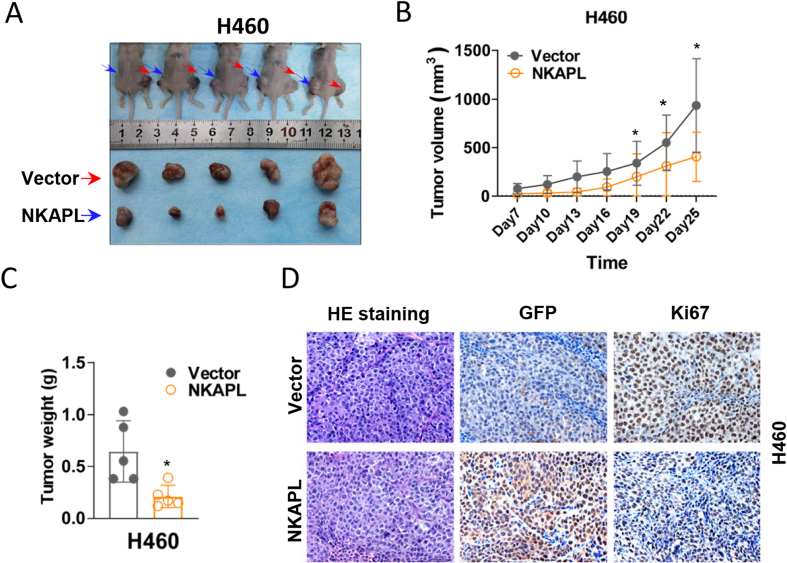


**figs5:**