# Supplementary material for: NKAPL suppresses NSCLC progression by enhancing the protein stability of TRIM21 and further inhibiting the NF-κB signaling pathway
Source: Genes Dis. 2025 Mar 11;12(5):101598. doi: 10.1016/j.gendis.2025.101598 (PMC12221590; doi:10.1016/j.gendis.2025.101598)
Supplement: Multimedia component 1 [file mmc1.docx]

**Supplemental Materials**

**
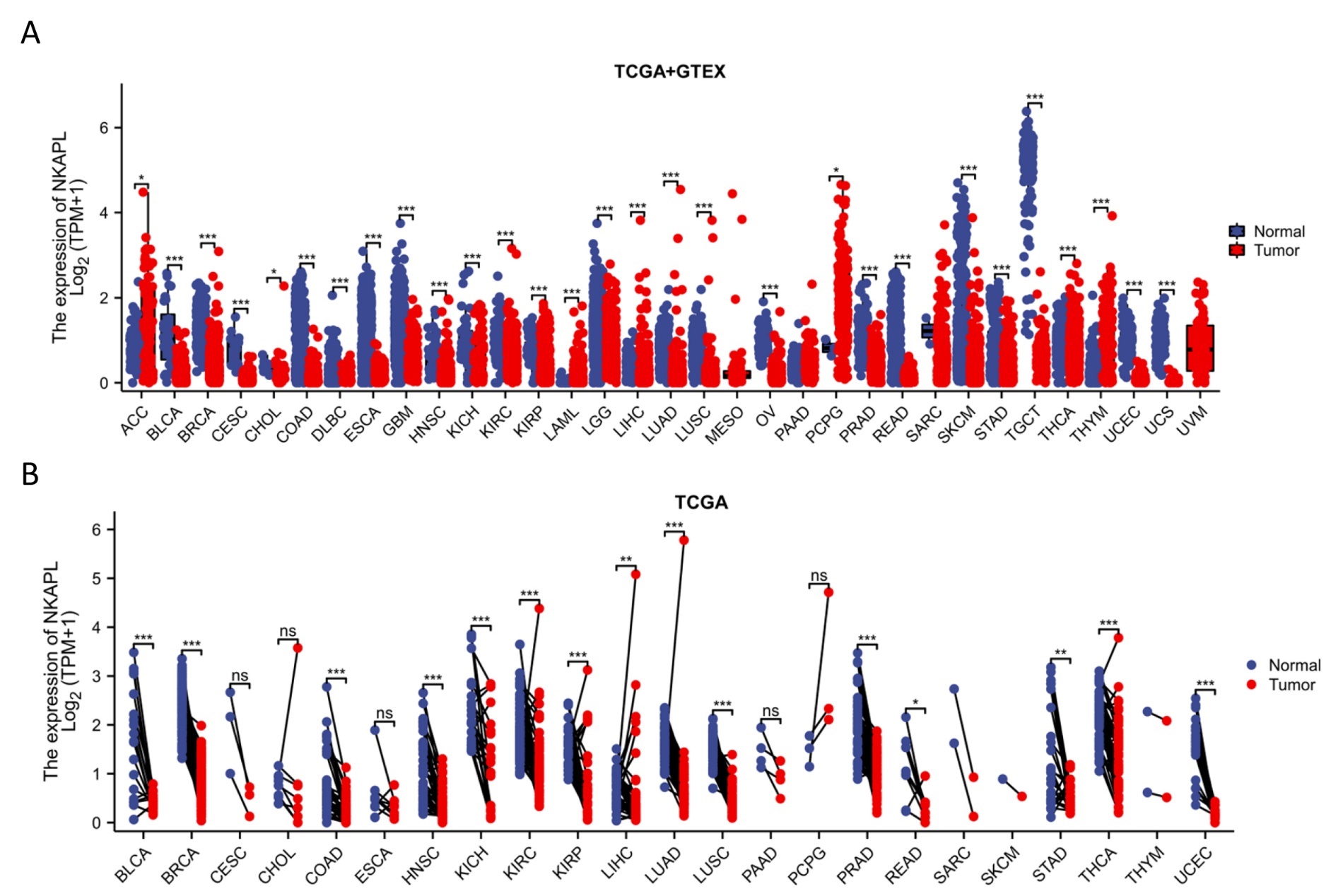
**

**Figure S.1 NKAPL expression in pan-cancer.**


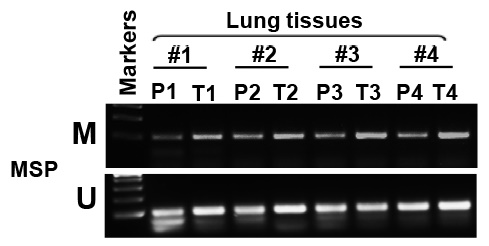
**(A).** Pan-cancer analysis of NKAPL expression in TCGA/GTEx. **(B).** Analysis of NKAPL expression in cancer tissues and adjacent paired normal tissues from TCGA. ns, p>0.05, *p<0.05, **p<0.01, ***p<0.001.

**Figure S2. Methylation of NKAPL in Lung tissues.**

NKAPL methylation was measured by MSP in primary NSCLC tissues and peritumoral lung tissue (n=4). T: non-small cell lung cancer tissue, P: peritumoral lung tissue.


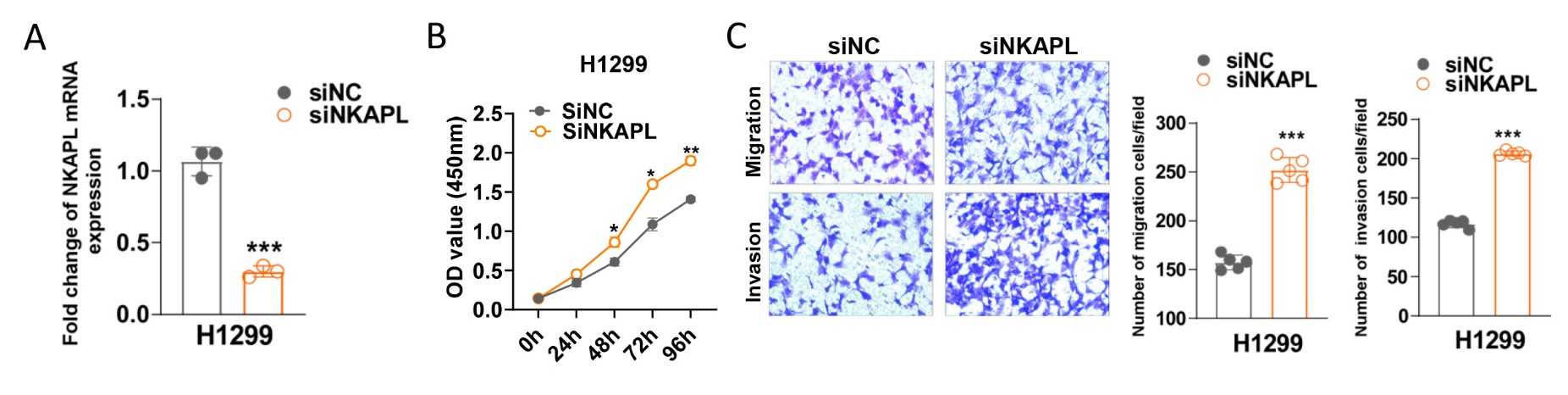


**Figure.S3 Knockdown of NKAPL promoted the proliferation, migration and invasion of NSCLC cells.**

**
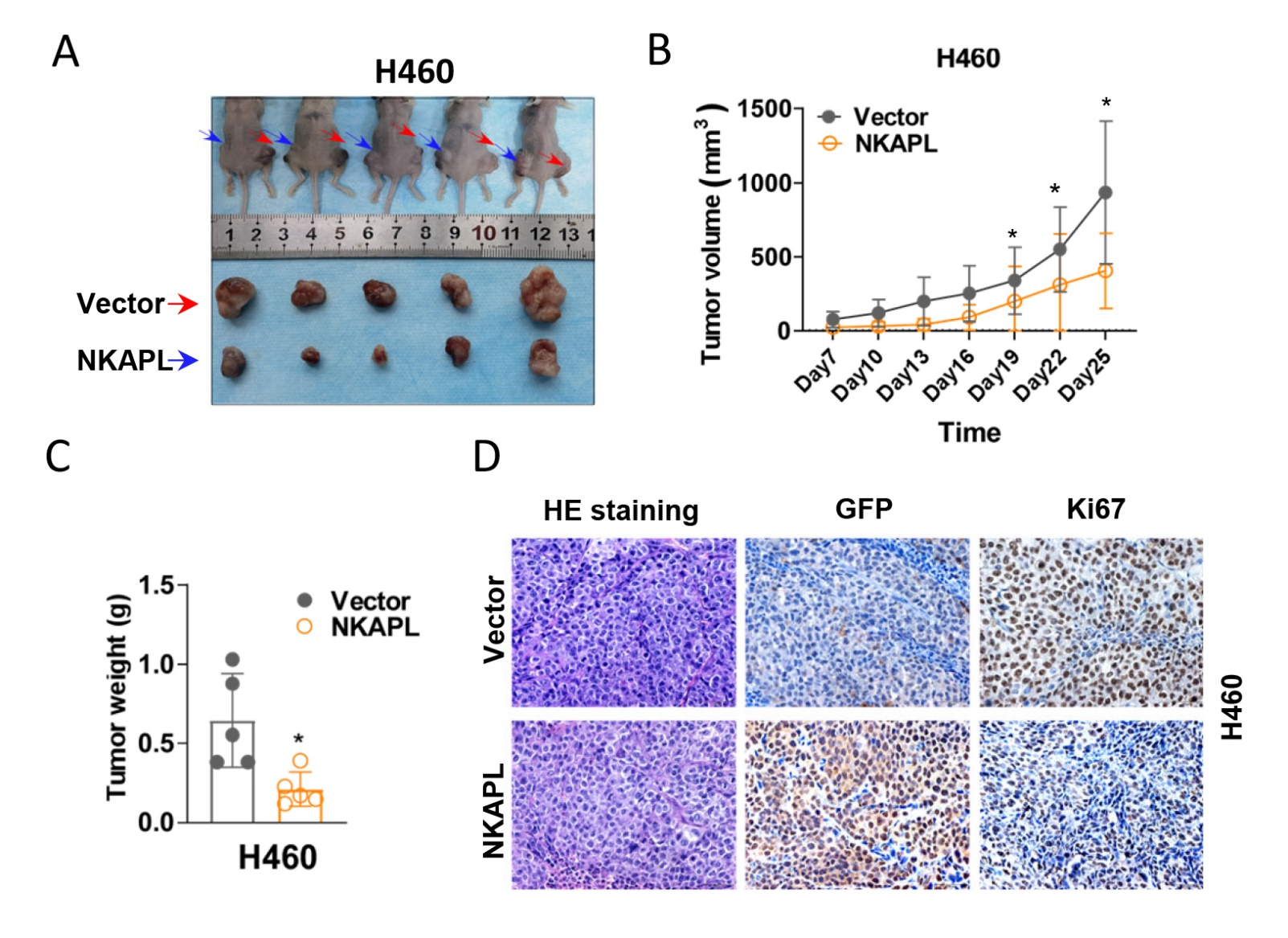
(A).** NKAPL siRNA was transfected into H1299 cells, and the expression levels of NKAPL were detected by qPCR. **(B).** Cell proliferation capacity was detected at the indicated time points by CCK8 assays. **(C).** Transwell assays were conducted to assess the invasion and migration capabilities of H1299-siNAKP and H1299-siNC cells. Data are presented as the mean ± SD of three independent experiments. *p<0.05, **p<0.01, ***p<0.001.

**Figure.S4 NKAPL suppressed NSCLC growth in vivo.**

**(A).** H460 cells stably expressing the vector and NKAPL-GFP were subcutaneously injected into BALB/c nude mice. Images of the subcutaneous tumors of the nude mice were captured. **(B-C)** The volume of the subcutaneous tumors in the nude mice was measured, and the tumors were weighed. **(D).** H&E staining and the expression changes of NKAPL, and Ki-67 in xenograft tumors were examined by IHC staining. The data are presented as the means ± SDs (n=3). *p < **
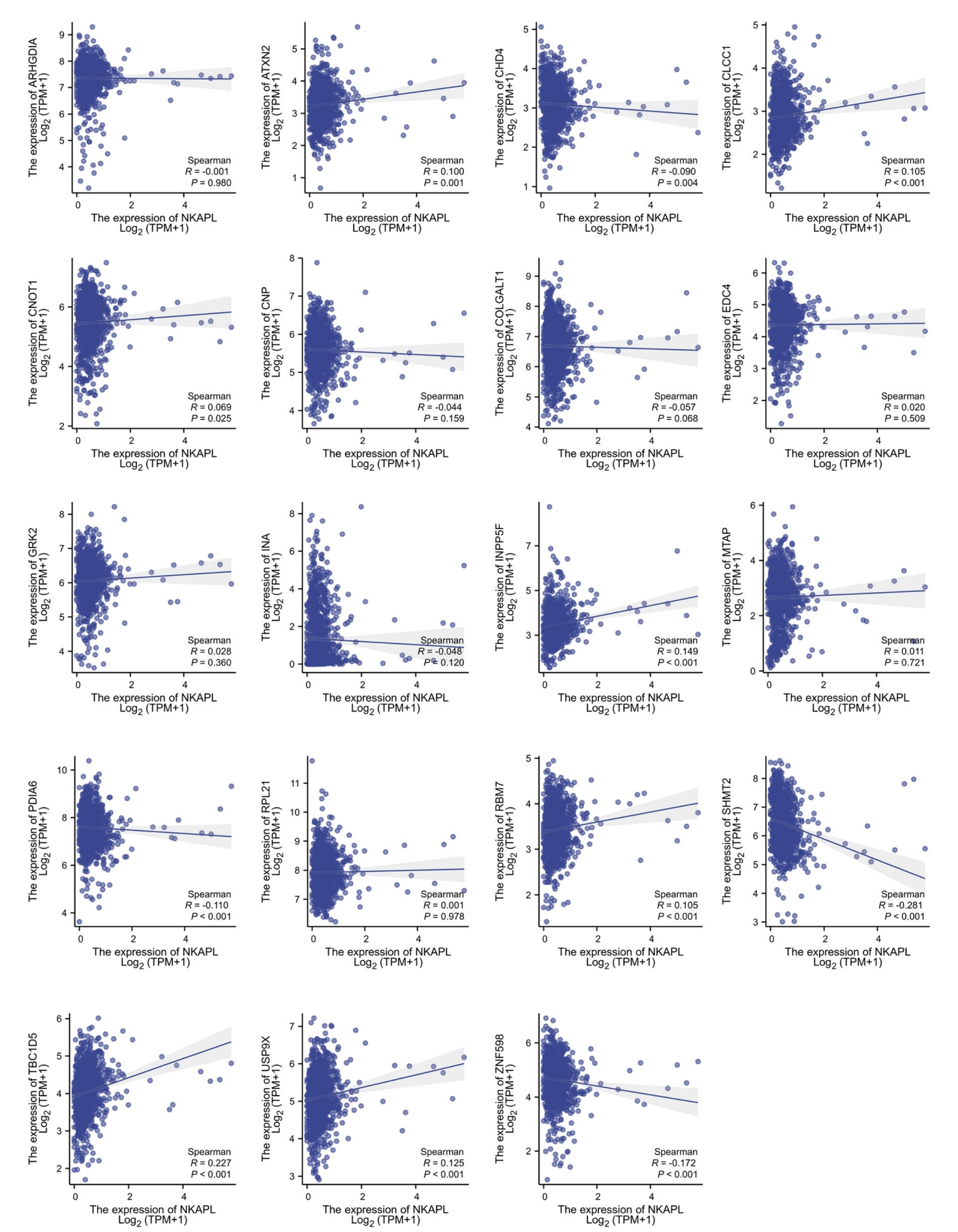
**0.05, **p < 0.01, ***p < 0.001.

**Figure.S5 Correlation between NKAPL expression and the top 20 NKAPL interacting protein coding genes in NSCLC.**
