# Supplementary material for: NKAPL suppresses NSCLC progression by enhancing the protein stability of TRIM21 and further inhibiting the NF-κB signaling pathway
Source: Genes Dis. 2025 Mar 11;12(5):101598. doi: 10.1016/j.gendis.2025.101598 (PMC12221590; doi:10.1016/j.gendis.2025.101598)
Supplement: Multimedia component 2 [file mmc2.docx]

Table S1

|  | Logistic Regression | SVM | MLP | GP | Random Forest |
| --- | --- | --- | --- | --- | --- |
| Score | 0.8673 | 0.7909 | 0.8664 | 0.8000 | **0.9027** |
